# A Case of Left Ventricular Noncompaction Presenting as Atrial Fibrillation

**DOI:** 10.7759/cureus.4309

**Published:** 2019-03-25

**Authors:** Anandbir S Bath, Sourabh Aggarwal, Vishal Gupta, Jagadeesh K Kalavakunta

**Affiliations:** 1 Internal Medicine, Western Michigan University Homer Stryker M.D. School of Medicine, Kalamazoo, USA; 2 Interventional Cardiology, Ascension Borgess Hospital, Kalamazoo, USA; 3 Cardiology, Ascension Borgess Hospital, Kalamazoo, USA

**Keywords:** cardiomyopathy, cardiac arrythmias, atrial fibrillation, noncompaction

## Abstract

Ventricular noncompaction is a rare congenital cardiomyopathy resulting from failure in cardiac embryogenesis. Both left and right ventricular involvement has been reported in nature. We present a case of a 69-year-old male presenting with atrial fibrillation complicated with in-hospital cardiac arrest in the setting of left ventricular noncompaction (LVNC). Our case demonstrates the fatal nature of this disease entity thus demanding a high index of suspicion for early detection and treatment.

## Introduction

Spongiform cardiomyopathy or left ventricular noncompaction (LVNC) is a rare congenital cardiomyopathy that can have varied presentation ranging from asymptomatic individuals to sudden cardiac death. It is one of the genetic cardiomyopathies, resulting from a failure in cardiac embryogenesis. This leads to unusual trabeculations in the left ventricular cavity which are most prominent in the midlateral-inferior parts of the left ventricle. Spongiform ventricle can serve as a foci for arrhythmias, which can be atrial or ventricular. Other complications include heart failure, systemic thromboembolism, and sudden cardiac death. We present a case of a 69-year-old man presenting with atrial fibrillation found to have LVNC in the setting of cardiac arrest.

## Case presentation

**Figure 1:**
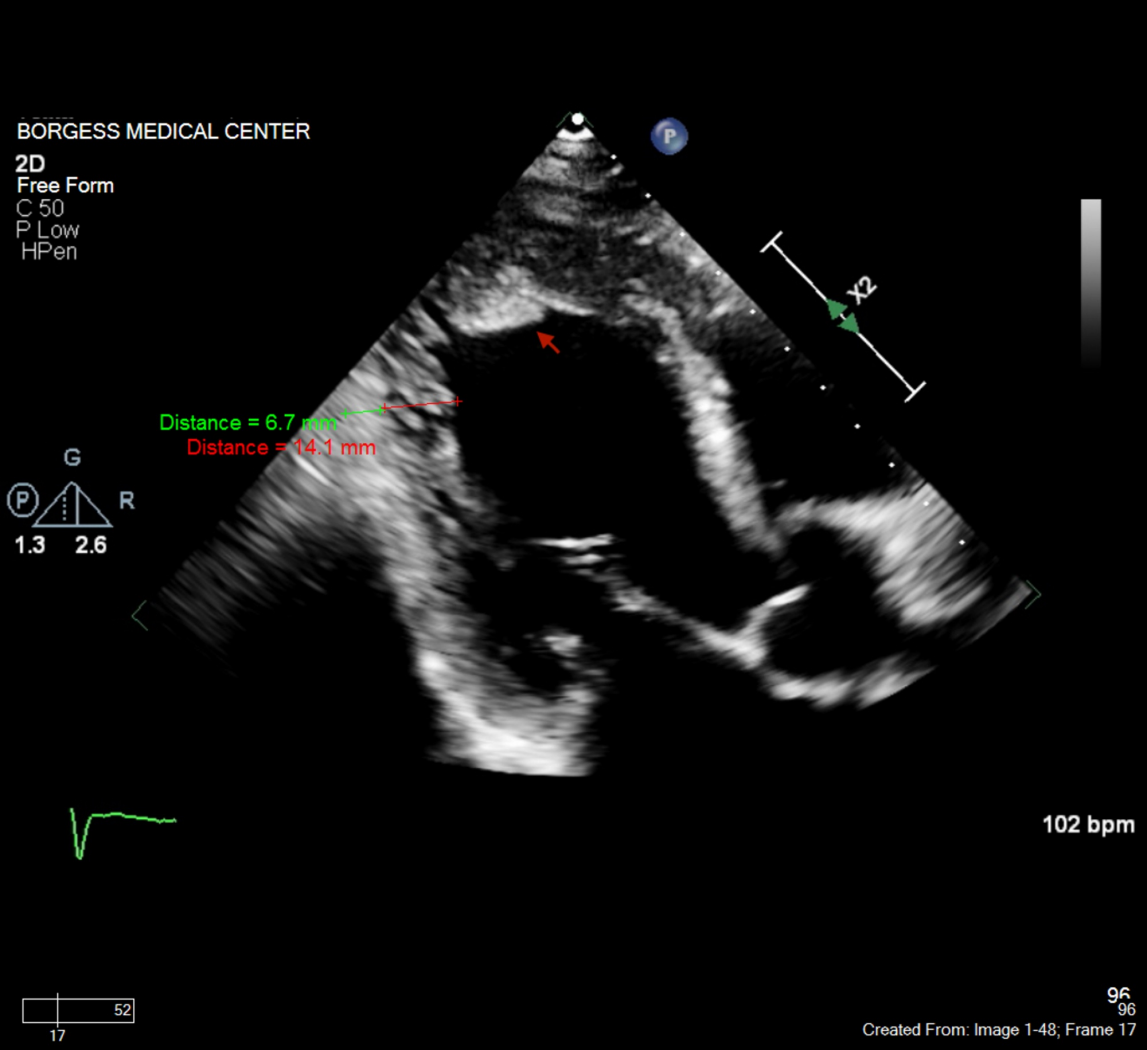
Transthoracic echocardiogram (TTE) showing left ventricular noncompaction with ratio of noncompacted to compacted myocardium of 2.1 The arrow depicts left ventricular thrombus.

A 69-year-old male with no past medical history presented with generalized fatigue and exertional shortness of breath. His initial vitals were significant for heart rate of 135 bpm with blood pressure of 108/70 mmHg. On examination, he was noted to be tachycardic with an irregular rhythm. He was also noted to have bibasilar rales and distended jugular vein. Electrocardiogram (EKG) confirmed atrial fibrillation with a rapid ventricular rate. His CHA_2_DS_2_-VASc (congestive heart failure, hypertension, age ≥75, diabetes mellitus, prior stroke or transient ischemic attack, vascular disease, age 65 to 74, female) score was 1 with HAS-BLED (hypertension, abnormal renal/liver function, stroke, bleeding history or predisposition, labile international normalized ratio, elderly (>65 years), drugs/alcohol concomitantly) score of 1 indicating need for anticoagulation with low risk for any major bleeding. He was started on intravenous heparin and diltiazem drip. His hospital course was complicated by cardiac arrest with pulseless electrical activity. He was successfully resuscitated with the return of spontaneous circulation after 8 minutes of cardiopulmonary resuscitation (CPR). Post-cardiac arrest transthoracic echocardiogram (TTE) revealed reduced ejection fraction of 10%-15% with features of LVNC. The ratio of noncompacted to compacted myocardium was 2.1 (Figure 1).

Also, there was evidence of left ventricular thrombus in the apical part as seen in Figure 1 arrow. Brain magnetic resonance imaging (MRI) done for neurological prognostication revealed multiple strokes and the decision was made to withdraw care.

## Discussion

Ventricular noncompaction is characterized by multiple trabeculations in the ventricular cavity accompanied with deep intratrabecular recesses which can involve both left and right ventricles. It results from failure of sinusoidal regression during the cardiac embryogenesis which leads to thin compacted epicardial layer and a spongy endocardial layer [1]. Though left ventricle is more commonly involved, cases of right ventricular involvement have also been reported [2]. Its prevalence is noted to be from 0.01%-0.04% in patients referred for echocardiography [3]. American Heart Association classified it as primary genetic cardiomyopathy, whereas the World Health Organization and European Society of Cardiology classify it as unclassified cardiomyopathy [4-5].

LVNC is the third most common primary cardiomyopathy in children with both sporadic and familial cases reported [6]. Multiple genetic mutations have been implicated in the pathogenesis. Though less prevalent in adults, its incidence is increasing with improved imaging modalities. Echocardiogram and cardiac magnetic resonance imaging are generally used to establish the diagnosis of this disease entity. An echocardiogram can reveal two layered myocardia with thick spongy endocardial layer and thin compacted epicardial layer. It usually involves apical and midlateral-inferior portions of the ventricle. Criteria for diagnosis include end systolic ratio of at least 2 of noncompacted to compacted myocardium with color Doppler evidence of deep intertrabecular recesses [7]. Myocardial fibrosis is observed on endomyocardial biopsy [8].

LVNC can have varied presentations ranging from asymptomatic individuals to sudden cardiac death. Sudden cardiac death is mostly attributed to ventricular arrhythmias as a result of LVNC [3,7]. Atrial fibrillation in the setting of non-compaction indicates poor prognosis and is the most common atrial arrhythmia seen in these patients. Its incidence is around 5%-29% according to major reports. Delayed conduction in the intertrabecular crypts coupled with subendocardial fibrosis in the noncompacted myocardium is thought to be the foci for arrhythmias. Other complications include progressive heart failure and systemic thromboembolism [9].

Management strategies include identifying the population at increased risk through genetic screening and prevention of complications. Treatment is usually symptomatic. Reduced systolic function warrants implantable cardioverter-defibrillator (ICD) placement [10]. Anticoagulation can be considered among patient with atrial fibrillation and a history of thromboembolism. Warfarin is most commonly used. Further research is needed to delineate the role of direct oral anticoagulants. Failure of medical therapy warrants an evaluation for heart transplantation.

Our patient presented with new-onset atrial fibrillation most likely with primary etiology of LVNC. Also, the apical thrombus seen on TTE could very well be the cause of his brain thromboembolism. Thus, entailing the fatal aspect of this disease entity. Furthermore, relatives of the patient were advised to undergo genetic testing due to the familial nature of the disease. If recognized in young age, advanced heart failure services can be involved earlier to devise a plan for management of the affected patients.

## Conclusions

Physicians should have a high index of suspicion for ventricular noncompaction as early detection and treatment can dramatically change the prognosis associated with this disease process. Definitive guidelines should be established for the screening of family members of the patients affected with spongiform cardiomyopathy. Once diagnosed, targeted therapies need to done to prevent the complications.

## References

[1] Jenni R, Oechslin EN, van der Loo B (2007). Isolated ventricular non-compaction of the myocardium in adults. Heart.

[2] Aggarwal S, Kalavakunta J, Gupta V (2016). A case of isolated right ventricle noncompaction with ST-elevation chest leads. Heart Views.

[3] Ritter M, Oechslin E, Sutsch G, Attenhofer C, Schneider J, Jenni R (1997). Isolated noncompaction of the myocardium in adults. Mayo Clinic Proc.

[4] Maron BJ, Towbin JA, Thiene G (2006). Contemporary definitions and classification of the cardiomyopathies. Circulation.

[5] Elliott P, Andersson B, Arbustini E (2008). Classification of the cardiomyopathies: a position statement from the European society of cardiology working group on myocardial and pericardial diseases. Eur Heart J.

[6] Andrews RE, Fenton MJ, Ridout DA, Burch M (2008). New-onset heart failure due to heart muscle disease in childhood: a prospective study in the United kingdom and Ireland. Circulation.

[7] Oechslin EN, Attenhofer Jost CH, Rojas JR, Kaufmann PA, Jenni R (2000). Long-term follow-up of 34 adults with isolated left ventricular noncompaction: a distinct cardiomyopathy with poor prognosis. J Am Coll Cardiol.

[8] Kalavakunta JK, Tokala H, Gosavi A, Gupta V (2010). Left ventricular noncompaction and myocardial fibrosis: a case report. Int Arch Med.

[9] Bhatia NL, Tajik AJ, Wilansky S, Steidley DE, Mookadam F (2011). Isolated noncompaction of the left ventricular myocardium in adults: a systematic overview. J Card Fail.

[10] Kobza R, Steffel J, Erne P (2010). Implantable cardioverter-defibrillator and cardiac resynchronization therapy in patients with left ventricular noncompaction. Heart Rhythm.

